# Overcoming the challenges associated with CD3+ T-cell redirection in cancer

**DOI:** 10.1038/s41416-020-01225-5

**Published:** 2021-01-19

**Authors:** Ajit Singh, Sundee Dees, Iqbal S. Grewal

**Affiliations:** 1 University of Pittsburgh School of Medicine, Pittsburgh, PA USA; 2 Janssen Biotherapeutics, The Janssen Pharmaceutical Companies of Johnson & Johnson, Spring House, PA USA

**Keywords:** Oncology, Translational research

## Abstract

The development of bispecific antibodies that redirect the cytotoxic activity of CD3+ T cells to tumours is a promising immunotherapeutic strategy for the treatment of haematological malignancies and solid cancers. Since the landmark FDA approval at the end of 2014 of the anti-CD3 × anti-CD19 bispecific antibody blinatumomab (Blincyto^®^) for the treatment of relapsed/refractory B-cell acute lymphoblastic leukaemia, ~100 clinical trials investigating the safety and efficacy of CD3+ bispecific T-cell redirectors for cancer have been initiated. However, despite early success, numerous challenges pertaining to CD3+ T-cell redirection in the context of cancer exist, including the recruitment of counterproductive CD3+ T-cell subsets, the release of systemic cytokines, the expansion of immune checkpoint molecules, the presence of an immunosuppressive tumour microenvironment, tumour antigen loss/escape, on-target off-tumour toxicity and suboptimal potency. The aim of the present review is to discuss novel approaches to overcome the key challenges associated with CD3+ bispecific T-cell redirection in order to achieve an optimal balance of anti-tumour activity and safety.

## Background

The development of antibody therapeutics has transformed the field of cancer immunotherapy. The first successful monoclonal antibody (mAb) therapy in oncology was rituximab (Rituxan^®^), a mouse–human chimeric immunoglobulin G1 (IgG1) mAb targeting the CD20 antigen on B cells for the treatment of non-Hodgkin’s lymphoma.^1^ Signalling-induced cell death and Fc-mediated effector functions, including antibody-dependent cellular cytotoxicity (ADCC) and complement fixation, have been identified as key processes that contribute to the multifactorial mechanism of the action of rituximab.^2,3^

**Fig. 1:**
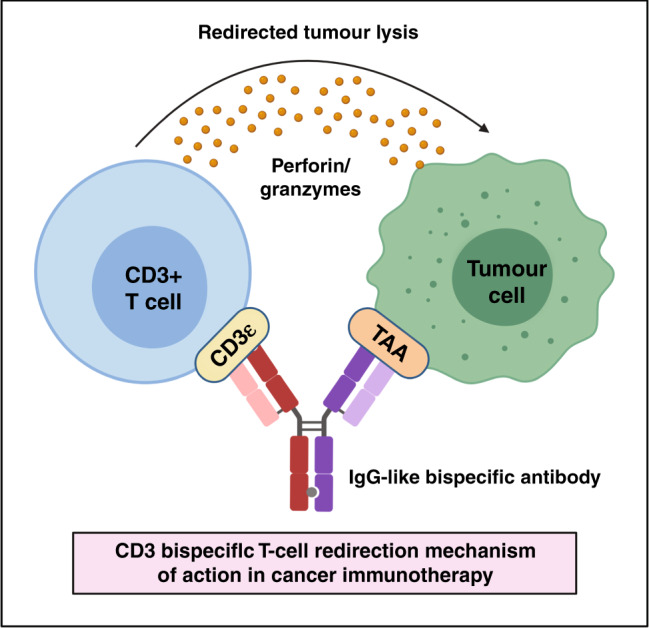
Mechanism of action of CD3+ bispecific T-cell redirection in cancer. The schematic depicts an IgG-like bispecific antibody simultaneously binding a tumour-associated antigen (TAA) on a cancer cell and CD3 epsilon on a T cell to redirect the cytotoxic activity of T cells to these tumour cells.

The concept of engineering mAbs directed against cancer antigens expressed on haematological malignancies was subsequently extended to solid cancers and was followed by the design of novel bispecific antibody formats capable of engaging multiple targets. CD3+ bispecific T-cell redirection antibody therapeutics, for example, mechanistically function by bridging T cells with cancer cells. In so doing, the cytolytic activity of CD3+ T cells can be redirected towards tumour cells to facilitate their elimination, independent of the usual requirement for the T cell to be bound to a major histocompatibility complex (MHC) molecule (MHC restriction)^4^ (Fig. 1). A major advantage associated with bispecific antibodies is that functional activity can be achieved, which would otherwise not be achievable in the context of monovalent antibody combinations. Nearly 25 years after its initial conceptualisation, the first bispecific CD3+ T-cell redirector, catumaxomab (Removab^®^), was approved by the European Union (EU) for the treatment of malignant ascites in 2009. However, this anti-CD3 × anti-epithelial cell adhesion molecule (EpCAM) antibody was shown to induce off-target hepatotoxicity in patients due to its binding to hepatic macrophages and was later withdrawn from the market due to commercial reasons.^5,6^

**Fig. 2:**
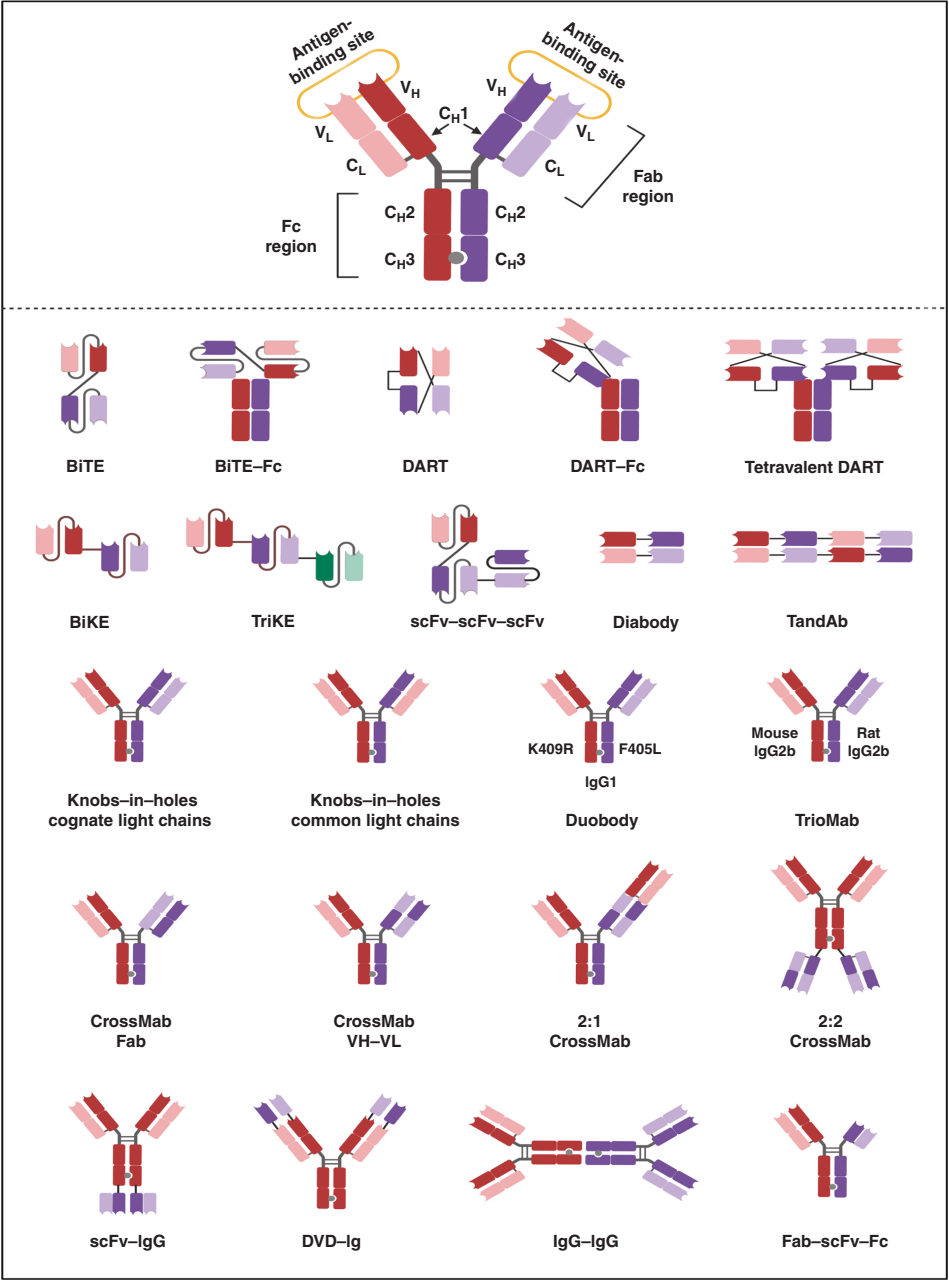
Bispecific antibody constructs for CD3+ T-cell redirection. A typical IgG-like bispecific antibody (shown) consists of two heavy chains and two light chains, subdivided into variable domains and constant domains. The Fab region (antigen-binding domain) and Fc region (effector-function domain) are indicated. V_H_ variable heavy, V_L_ variable light, C_H_ constant heavy, C_L_ constant light. Various bispecific antibody formats exist for CD3+ T-cell redirection: bispecific T-cell engager (BiTE), BiTE-Fc; dual-affinity re-targeting (DART), DART-Fc; tetravalent DART; bispecific killer cell engager (BiKE); tri-specific killer cell engager (TriKE); single-chain variable fragment (scFv)-scFv-scFv; diabody; tandem diabody (TandAb); knobs-in-holes cognate light chains; knobs-in-holes common light chains; DuoBody; TrioMab; CrossMab Fab; CrossMab VH-VL; 2:1 CrossMab; 2:2 CrossMab; scFv-IgG; DVD-Ig; IgG-IgG; and Fab-scFv-Fc. DuoBody bispecific antibodies are formatted on an IgG1 backbone and are generated by controlled Fab arm exchange of complementary C_H_3 domain mutations that promote heterodimerisation (K409R refers to a lysine (K) to arginine (R) amino acid substitution; F405L refers to phenylalanine (F) to leucine (L) amino acid substitution).

The Food Drug Administration (FDA) subsequently approved the anti-CD3 × anti-CD19 bispecific T-cell engager blinatumomab (Blincyto^®^) for the treatment of patients with Philadelphia chromosome-negative B-cell acute lymphoblastic leukaemia (ALL).^7^ FDA approval of this drug was based on the results of the multicentre, open-label, single-arm Phase 2 BLAST clinical trial (NCT01207388), in which 78% of patients with B-cell ALL achieved a complete minimal residual disease (MRD) response after just one cycle of treatment. Compared with MRD non-responders, patients who achieved complete MRD responses also demonstrated longer overall survival (12.5 versus 38.9 months; *P* = 0.002) and relapse-free survival (5.7 versus 23.6 months; *P* = 0.002).^8^ The unprecedented clinical success of blinatumomab has since laid the foundation for the development of CD3+ T-cell-engaging bispecific antibodies. Although blinatumomab is structurally formatted as a bispecific T-cell engager (BiTE)^®^, additional bispecific antibody designs have been described for CD3+ T-cell redirection (Fig. 2). Next-generation CD3+ bispecific T-cell redirectors are under preclinical and clinical investigation. To date, ~100 clinical trials have been initiated for CD3+ bispecific T-cell redirection in cancer.^5,9–11^

Blinatumomab is now approved for the treatment of patients with Philadelphia-negative and -positive relapsed/refractory ALL, as well as those patients with ALL who are in remission but show signs of MRD; however, its use is associated with elevated levels of cytokine release and neurotoxicity, although these toxicities can be clinically managed.^10^ Indeed, CD3+ T-cell redirection has been reported to be associated with a number of challenges that might limit its use or reduce its anti-tumour efficacy, including the recruitment of counterproductive CD3+ T-cell subsets, dose-limiting cytokine storm, the presence of an immunosuppressive tumour microenvironment (TME), T-cell dysfunction and exhaustion due to expression of immune checkpoint molecules, tumour antigen escape, on-target off-tumour toxicity and suboptimal potency, all of which will be outlined in this article (Table 1). Continued progress in the clinic and momentum in the field of cancer immunotherapy will therefore rely on addressing these major obstacles, and therefore novel strategies to overcome the challenges associated with the use of T-cell redirection are also described herein.

**Table 1:** Mitigation strategies to overcome the challenges associated with CD3+ bispecific T-cell redirection in cancer.

Challenges associated with CD3+ T-cell redirection in cancer	Resulting consequences	Mitigation strategies
Recruitment of pan T-cell populations	Activation/redirection of counterproductive CD3+ T-cell subsets (e.g., Tregs, naive/exhausted T cells, CD4+ T cells that secrete excessive amounts of cytokines)	Selective redirection of CD8+ cytotoxic T lymphocytes or CD8+ tissue-resident memory T cells
Cytokine release syndrome	Cytokine storm (life-threatening immune-related adverse event); dose-limiting; efficacy-limiting; narrow therapeutic window	Pharmacological intervention with IL-6R antagonists, TNF-α blockers, or corticosteroids; quantitative cytokine modelling to improve clinical dosing strategy; sequence-based antibody discovery to decouple cytokine release from anti-tumour activity
Expression of immune checkpoint molecules	T-cell dysfunction and exhaustion lead to suppressed anti-tumour immunity	Combination therapy with co-inhibitory receptor antagonists (e.g., anti-PD-1, anti-CTLA-4) or co-stimulatory receptor agonists (e.g., anti-4-1BB, anti-CD28)
Immunosuppressive tumour microenvironment	Hostile TME components (e.g., stromal cells, immune cells, cytokines, soluble factors) contribute to suppression of anti-tumour immune response	Combination therapy with IL-10 inhibitors, adenosine receptor antagonists, or VLA4 inhibitors; oncolytic adenoviruses armed with CD3 redirectors that target immunosuppressive stromal cells
Tumour antigen escape	Resistance to CD3+ T-cell redirection	Development of CD3+ T-cell redirectors with dual tumour antigen targeting
Lack of tumour antigen selectivity	On-target off-tumour toxicity	Protease-activated T-cell engagers; hemibodies; development of a multivalent avidity-based antigen-binding strategy to generate bispecific antibodies that selectively redirect T cells towards tumour cells while avoiding healthy tissue
Suboptimal potency	Diminished anti-tumour immune response	Consideration of target antigen copy number and size; modulation of binding domain positioning (e.g., epitope distance) and valency

## Recruitment of pan T-cell populations

CD3+ bispecific T-cell engagers function by redirecting all T-cell populations towards cancer cells to facilitate efficient tumour cell killing. However, several disadvantages are associated with polyclonal T-cell activation and redirection, including the recruitment of ‘counterproductive’ CD3+ T-cell subsets, such as naive/exhausted T cells, regulatory T (T_REG_) cells and other CD4+ T-cell populations.^12–14^ In fact, resistance to blinatumomab has been attributed to the activation and recruitment of CD3+ CD4+ CD25^hi^ FoxP3^+^ T_REG_ cells, which have been shown to suppress T-cell proliferation and hamper CD8+-mediated tumour cell lysis, ultimately compromising the efficacy of blinatumomab treatment in patients with precursor B-cell ALL.^15^ In addition to T_REG_ cells, excessive recruitment of other CD4+ T-cell populations, including the helper T (T_H_) cell subsets TH_1_ and TH_17_, can be detrimental because these cells can rapidly secrete a milieu of cytokines leading to severe immune-related adverse events such as cytokine storm, which is outlined below.^16,17^

### Improving the selectivity of T-cell-engaging bispecific antibodies

The selective recruitment of CD8+ cytotoxic T lymphocytes (CTLs)—so-called foot soldiers of the immune system—to tumour cells represents a more productive approach to drive potent anti-tumour immune responses compared with non-specific pan CD3+ T-cell redirection, as professional CD8+ CTLs are equipped with perforin/granzymes and can initiate killing immediately upon crosslinking with tumour cells.^18,19^ However, a bispecific antibody targeting the CD8 co-receptor and prostate stem cell antigen (PSCA) constructed to test this approach was able to efficiently engage only pre-activated CD8+ T cells and redirect them for tumour cell lysis—freshly isolated CD8+ T cells could not be activated/redirected—thus signifying the importance of both pre-activation and co-ligation in inducing selective tumour killing.^17^ The tissue-resident memory (TRM) CD8+ CD69+ CD103+ T-cell phenotype has also emerged as an important T-cell population for selective retargeting to tumour cells, as this memory CD8+ T-cell subset expresses high levels of cytotoxic molecules and is associated with a good clinical outcome in cancer.^20^ Menares et al.^21^ also demonstrated, in 2019, a crosstalk between TRM T cells and dendritic cells that leads to an amplified CD8+ CTL-mediated anti-tumour immune response. In view of these results, research efforts are underway to improve the selectivity of T-cell-engaging bispecific antibodies in favour of more potent tumour cell killing accompanied by reduced toxicity.

## Cytokine release syndrome

Cytokine release syndrome is a severe immune response that is characterised by the rapid, systemic release of pro-inflammatory cytokines, including interleukin (IL)-6, IL-10, tumour necrosis factor-α (TNF-α) and interferon-γ (IFN-γ). Elevated levels of these core cytokines induce immune cells to produce excessive amounts of additional cytokines, creating an amplified phenomenon referred to as a ‘cytokine storm’.^22,23^ This immune-related adverse event is frequently observed in response to chimeric antigen receptor (CAR)-T cell therapy or T-cell redirection therapy and presents a major challenge in the context of CD3+ T-cell redirection because the infused bispecific antibodies indiscriminately bind to all available T cells, leading to a massive release of cytokines. Indeed, blinatumomab and two FDA-approved CAR-T cell therapies, tisagenlecleucel (Kymriah) and axicabtagene ciloleucel (Yescarta), have been shown to induce cytokine release syndrome in patients with leukaemia and lymphoma.^24^ Clinical manifestations of cytokine release syndrome include fever, fatigue, myalgia, respiratory distress, capillary leakage, vasodilatory shock and eventual organ system failure.^22^ CD19-CAR-T cells armed with anti-CD3 × anti-HER2 or anti-CD3 × anti-epidermal growth factor receptor (EGFR) bispecific antibodies have been developed to address the cytokine release syndrome.^25^ This approach is self-limiting and holds potential for reducing the risk of the occurrence of a cytokine storm as the amount of bispecific antibody on the bispecific antibody-armed CAR-T cell decreases after multiple cell divisions.

### Managing the cytokine release syndrome using pharmacological approaches

A number of pharmacological approaches have been investigated to overcome the cytokine release syndrome associated with T-cell redirection therapies. Monocyte activation mediated by TNF-α generated by T-cell triggering has been demonstrated to be a primary source of systemic cytokine release after treatment with anti-CD3 × anti-HER2 bispecific antibodies and, interestingly, TNF-α blockade alone was sufficient to impair cytokine release without affecting the cytotoxic activity of the T-cell-redirecting antibodies.^26^ Furthermore, administration of the immunosuppressant dexamethasone prior to treatment with an anti-CD3 × anti-glypican 3 (GPC3) bispecific antibody led to significant inhibition of cytokine release without compromising the anti-tumour activity in a humanised mouse model.^27^ In another study, pre-treatment with the anti-IL-6 receptor antibody tocilizumab prior to administration of an anti-CD3 × anti-prostate-specific membrane antigen (PSMA) bispecific antibody attenuated the cytokine release syndrome while maintaining therapeutic activity in patients with castration-resistant metastatic prostate cancer.^28^ However, in this same study, dexamethasone treatment prior to bispecific antibody therapy was shown to negatively impact anti-tumour efficacy, as evidenced by suppression of T-cell proliferation and inhibition of tumour lysis.^28^ Similarly, the data from a Phase 1 clinical trial showed that dexamethasone treatment did not increase the maximum tolerated dose of an anti-CD3 × anti-carcinoma embryonic antigen (CEA) bispecific antibody (NCT01284231). Taken together, the results of these studies suggest that co-administration of CD3+ T-cell-redirecting therapies with steroids, anti-TNF-α blockers or IL-6 receptor targeting antibodies might, in certain circumstances, help to mitigate the cytokine storm associated with T-cell redirection.^29^ The outcomes of such combinatorial approaches are context-dependent and could be influenced by the specific mechanism of a given T-cell redirector. Thus, a deeper mechanistic understanding of the relationship between T-cell activation and the cytokine storm is warranted. The identification of clinical biomarkers that are predictive of a cytokine release syndrome will aid in the future development of novel therapeutic agents and approaches that are capable of mitigating the cytokine storm in the context of T-cell redirection. Of note, regression modelling has been used to identify a cytokine signature in patients that is predictive of the development of the cytokine release syndrome after CAR-T cell treatment.^30^

### Novel strategies to mitigate the cytokine release syndrome

Although the cytokine release syndrome has the potential to be managed in part as described above, research efforts are focused on developing novel strategies to mitigate the syndrome to increase the therapeutic window for T-cell-engaging therapies. The unprecedented cytokine storm observed in six healthy volunteers within 90 min of receiving TGN1412, a super-agonist antibody targeting the T-cell costimulatory receptor CD28, sent shockwaves throughout the scientific community as this life-threatening inflammatory response was not predicted by preclinical toxicology models.^24,31^ Preventing future occurrences of this magnitude in other T-cell-based immunotherapies quickly became a priority. Since this devastating Phase 1 clinical trial, different clinical dosing regimens for T-cell-engaging therapeutic antibodies have been considered. A priming dose strategy has been tested in the clinic to mitigate the toxicities induced by the cytokine release syndrome—this approach involves administration of a lower initial dose followed by a higher maintenance dose (NCT02152956).^32,33^ As the methodology of selecting an optimal priming dosing regimen is empirical by nature, quantitative cytokine modelling was later introduced to improve the design of clinical dosing strategies to achieve efficacy while minimising cytokine release syndrome-related toxicities. Indeed, a novel pharmacokinetic–pharmacodynamic (PK–PD) model developed in 2019 simulates the cytokine release profiles in response to treatment with T-cell-redirecting therapies.^33^ A quantitative systems pharmacology model was subsequently established to enable the accurate prediction of safety- and efficacy-related biomarkers to reduce the risk of cytokine storm with the anti-CD3 × anti-CD20 bispecific antibody mosunetuzumab in non-Hodgkin lymphoma (NCT02500407).^34^

Although quantitative modelling has been used to determine the optimal dosing to mitigate the cytokine release syndrome associated with current T-cell-engaging antibodies, next-generation CD3+ T-cell-recruiting bispecific antibodies have been engineered to directly address the challenge of limiting the dose to prevent cytokine release syndrome. Initial anti-CD3+ antibody generation efforts were often biased towards high-affinity CD3 binders, which elicited not only potent tumour cell killing, but also high levels of cytokine release. The identification of naturally occurring anti-CD3 antibodies with varying binding affinities and T-cell activation profiles soon became a priority. A next-generation sequence (NGS)-based antibody discovery platform established in 2019 to identify anti-CD3 antibodies from humanised rats that bind to multiple CD3 epitopes with a diverse range of binding strengths was used to identify lead anti-CD3-binding domains, which were then formatted as bispecific antibodies.^35^ In particular, the anti-CD3 × anti-BCMA bispecific T-cell redirector that emerged from this antibody screen was shown to elicit robust tumour antigen-specific killing in a mouse xenograft model with almost undetectable cytokine release. Importantly, this work provides proof of concept for uncoupling of tumour cell cytotoxicity from cytokine release, thus relieving the dose constraints associated with T-cell engagers and broadening the therapeutic index. Collectively, these results show that PK–PD modelling and sequence-based antibody discovery strategies can be used to mitigate cytokine release syndrome-related toxicities from T-cell-redirecting antibody therapeutics.

Additional next-generation T-cell-recruiting bispecific antibodies have been described, including an elegant body of work that demonstrated the use of bispecific antibodies to retarget ex vivo expanded T cells to solid and haematological malignancies such as non-Hodgkin’s lymphoma, multiple myeloma, pancreatic cancer, breast cancer and prostate cancer. These armed T-cell infusions not only show preclinical and clinical anti-tumour efficacy, with or without stem cell transplantation, but, importantly, avoid cytokine release syndrome. The lack of apparent dose-limiting toxicity provides a rationale for the use of bispecific antibody-armed activated T-cell infusions as a strategy to overcome the challenge of cytokine release syndrome.^36–41^

## Expression of immune checkpoint molecules

Inhibitory checkpoint molecules and stimulatory checkpoint molecules function alongside each other to ensure that the immune system can mount a response against ‘foreign’ bodies but that this response is controlled, in terms of type, magnitude and duration, in order to maintain self-tolerance and prevent autoimmunity. It is well established that inhibitory molecules expressed on the surface of T cells or tumour cells can promote T-cell dysfunction and exhaustion, leading to a suppressed anti-tumour immune response. Examples of such co-inhibitory immune checkpoint molecules include programmed death-1 (PD-1), programmed death-ligand 1 (PD-L1), cytotoxic T lymphocyte-associated antigen-4 (CTLA-4), T-cell immunoglobulin and mucin domain-containing protein 3 (TIM3), lymphocyte-activation gene 3 (LAG3) and T-cell immunoreceptor with immunoglobulin and ITIM domains (TIGIT).^42^ Enrichment of immune checkpoint receptor expression was observed in CD8+ CD69+ CD103+ human T cells with a TRM phenotype, and anti-PD-1 treatment led to a significant expansion of this T-cell population in melanoma patients.^43^ Moreover, anti-PD-1 therapy was shown to enhance the infiltration of tissue-resident CD8+ memory T cells in a mouse model of melanoma.^44^

### CD3+ bispecific T-cell redirection combined with immune checkpoint co-inhibitors

Combinatorial immunotherapy approaches have been designed to counteract the mechanisms of T-cell dysfunction/exhaustion using inhibitory checkpoint-blocking antibodies to improve the clinical outcomes of individuals receiving CD3+ bispecific T-cell redirection therapies. Interestingly, resistance to blinatumomab treatment has been attributed to an increase in PD-L1 expression on CD19+ leukaemic cells.^45^ Since this discovery, a number of clinical trials evaluating the safety and efficacy of blinatumomab in combination with pembrolizumab (anti-PD-1 therapy) for the treatment of lymphoma or leukaemia have been initiated (NCT03160079, NCT03605589, NCT03340766, NCT03512405). Furthermore, the addition of the dual immune-checkpoint blockade to blinatumomab therapy is under investigation in the clinic (e.g., a Phase 1 trial of blinatumomab and nivolumab (an anti-PD-1 antibody) with or without ipilimumab (an anti-CTLA-4 antibody) for the treatment of leukaemia is underway (NCT02879695)). A bifunctional checkpoint inhibitory T-cell engager (CiTE) that redirects T cells to CD33 (expressed on acute myeloid leukaemia (AML) cells), combined with the extracellular domain of PD-1 to bind weakly to, but inhibit engagement of PD-L1, demonstrated early preclinical success in mitigating adaptive immune escape in patients with AML.^46^ Notably, anti-CD3 × anti-PD-1 × anti-HIV and anti-CD3 × anti-TIGIT × anti-HIV trifunctional T-cell engagers have demonstrated efficacy in enhancing CD8+ T-cell effector functions in SHIV-infected rhesus macaques;^47^ this model can probably be applied in the context of cancer. Immune checkpoint inhibition coupled with CD3+ bispecific T-cell redirection has also been examined in solid tumours. The safety and efficacy of MGD007 (anti-CD3 × anti-gpA33 bispecific antibody) in combination with MGA012 (an anti-PD-1 antibody) is currently under clinical investigation in a Phase 1/2 trial for metastatic colorectal cancer (NCT03531632). A quantitative systems pharmacology model and biomarker strategy revealed that combination therapy comprising an anti-CD3 × anti-CEA bispecific antibody with anti-PD-L1 treatment led to an enhancement of anti-tumour activity in patients with colorectal cancer.^48^

### CD3+ bispecific T-cell redirection combined with immune checkpoint co-stimulators

Although antagonising co-inhibitory receptors on T cells has proven to be a promising approach for enhancing the anti-tumour activity of CD3+ T-cell redirectors, there is also evidence that agonistic antibodies that are capable of activating co-stimulatory receptors on T cells, including members of the tumour necrosis factor receptor (TNFR) superfamily (e.g., 4-1BB, OX40, GITR, CD27) or immunoglobulin superfamily (e.g., CD28, ICOS), can be used to boost immune responses to CD3+ T-cell-engaging therapies. Notably, an agonistic anti-4-1BB antibody was shown to restore the functionality of exhausted CD8+ tumour-infiltrating lymphocytes in a mouse model of melanoma.^49^ Likewise, stimulation of 4-1BB signalling with an agonistic anti-4-1BB antibody led to the prolonged persistence of cytotoxic T lymphocytes and their enhanced function in a B16F10-OVA melanoma mouse model.^50^ Anti-4-1BB co-stimulation was also demonstrated to enhance the anti-tumour activity of an anti-CD3 × anti-PSMA bispecific antibody both in vitro and in a syngeneic mouse model of prostate cancer.^51^ Furthermore, combination therapy of fibroblast activation protein (FAP)-4-1BBL with a CEA-targeted T-cell bispecific antibody plus CD19-4-1BBL with a CD20-targeted T-cell bispecific antibody led to significant tumour regression in mouse models and intratumoural accumulation of activated CD8+ cytotoxic T cells. Mechanistically, in the presence of T-cell receptor signalling, tumour antigen-targeted 4-1BB agonists induce 4-1BB stimulation in response to crosslinking with tumour antigen-expressing cells without relying on FcγR-mediated crosslinking.^52^ As well as 4-1BB, other co-stimulatory molecules such as CD28 have been shown to improve anti-tumour immune responses to CD3+ T-cell-redirecting antibodies. Treatment of cancer cells with anti-CD3 × anti-tumour antigen and anti-CD28 × anti-tumour antigen cross-interacting bispecific antibodies led to enhanced tumour cell-dependent T-cell activation in vitro.^53^ Moreover, anti-CD28 × anti-PSMA and anti-CD28 × anti-mucin 16 (MUC16) bispecific antibodies were shown to potentiate in vivo anti-tumour activity of CD3+ bispecific T-cell redirectors in humanised immunocompetent mouse models of prostate and ovarian cancer, respectively.^54^ Importantly, combination therapy did not elicit toxicity in cynomolgus monkeys in vivo. These results indicate that the expression profile of co-stimulatory (and co-inhibitory) molecules on patient T cells or tumour cells can inform of potential combination therapies with CD3+ T-cell redirectors.

## Immunosuppressive TME

Fundamental differences exist in the disease phenotype of haematological malignancies and solid cancers, an obvious one being the highly immunosuppressive TME that is a characteristic feature of solid tumours. Major components of the TME of solid tumours include anti-tumour immune cells together with stromal cells, such as cancer-associated fibroblasts (CAFs), T_REG_ cells, myeloid-derived suppressor cells (MDSCs) and tumour-associated macrophages (TAMs), that function in suppressing anti-tumour immunity.^55–57^ Immunosuppressive cytokines and soluble factors including adenosine, IL-10, transforming growth factor β (TGF-β), and indoleamine 2,3-dioxygenase (IDO) also reside in the hostile TME.

### Targeting the hostile TME in conjunction with CD3+ bispecific T-cell redirection

Disrupting the reactive stroma that surrounds solid tumours represents a viable approach to increase the anti-tumour activity of T-cell-engaging bispecific antibody therapeutics. In fact, adenosine and IL-10 have been demonstrated to directly inhibit CD8+ T-cell function, thus providing a rationale for combination therapy of CD3+ T-cell redirectors with adenosine receptor antagonists or IL-10 inhibitors.^58,59^ Furthermore, an oncolytic adenovirus armed with an anti-CD3 × anti-FAP-α bispecific antibody redirected the cytotoxic activity of T cells towards CAFs residing in the stroma, thereby eliminating these stromal cells and facilitating the penetration and spread of the adenovirus to ultimately enhance the viral oncolysis of cancer cells.^60^ In addition, anti-CD3 × anti-CD206 and anti-CD3 × anti-folate receptor β (FRβ) T-cell engagers demonstrated preferential T-cell-mediated killing of M2 polarised TAMs in vitro,^61^ and localisation of the expression of these T-cell engagers to tumours using the oncolytic virus enadenotucirev caused a reversal of the immunosuppressive TME of solid tumours via targeted TAM depletion and potent cancer cell cytotoxicity.

As with solid cancers, the TME of haematological malignancies also presents a challenge to T-cell-redirecting therapies. The bone marrow niche in haematological cancers is a specialised microenvironment that contains soluble growth factors necessary for the survival of resident cancer stem cells (CSCs). The persistence of malignant progenitor cells such as CSCs in the bone marrow is linked to MRD and is a predominant source of resistance to antibody therapeutics. Nair-Gupta et al.^62^ demonstrated in 2020 that co-culture of bone marrow stromal cells with AML or multiple myeloma cells prevented the lysis of tumour cells by anti-CD3 × anti-CD123 or anti-CD3 × anti-BCMA bispecific T-cell redirectors in vitro and in vivo. Strikingly, however, inhibition of the integrin VLA4, which mediates interactions between tumour and stromal cells, not only reversed this stromal-mediated immunosuppression, but restored the sensitivity of leukaemic and myeloma tumour cells to CD3+ bispecific T-cell redirectors, as evidenced by robust in vitro and in vivo anti-tumour activity. Thus, targeting the hostile TME in combination with CD3+ bispecific T-cell redirection therapies constitutes a promising approach to overcome stroma-mediated immunosuppression and elicit greater anti-tumour immune responses.

### Physiologically relevant models to assess combinatorial approaches

Given the complexity of the TME, designing optimal preclinical models that recapitulate human physiology and the disease phenotype remains a priority—the development of preclinical models that translate to human immunity was described by Hegde et al.^63^ as being a top key challenge in cancer immunotherapy. Three-dimensional multicellular tumour spheroids and ‘tumour on a chip’ microfluidics devices are examples of next-generation platforms that have been developed to increase preclinical-to-clinical translation of drug candidates.^64,65^ Such complex in vitro models are engineered to incorporate extracellular matrix components, soluble factors, tissue barriers, hypoxia gradients, interstitial pressure and vascular perfusion to recreate organ-specific architecture. Stromal cells, immune cells and endothelial cells can also be integrated into these micro-physiological tumour models to enable a deeper understanding of the interactions between cancer cells and their surrounding TME. Indeed, lysis mediated by T-cell-receptor (TCR)-engineered T cells of hepatocellular carcinoma cells embedded in a 3D collagen microenvironment was demonstrated using a microfluidics platform.^66^ Moreover, proof of concept was established for the assessment of tumour cell cytotoxicity by TCR-engineered T cells under hypoxic and inflammatory conditions in the microfluidics device. Physiologically relevant complex in vitro models can therefore represent powerful preclinical tools that can be used to evaluate such combinatorial immunotherapies to better predict translational success.

## Tumour antigen escape and lack of tumour antigen specificity

The loss of expression of tumour antigens is another factor that can compromise the efficacy of CD3+ bispecific T-cell redirection therapies. For example, 10–20% of patients with B-cell ALL have been shown to experience resistance to blinatumomab treatment due to CD19 antigen loss.^67^ The emergence of CD19^−^ leukaemic blasts during blinatumomab therapy has been mechanistically linked to disruptions in the trafficking of CD19 to the membrane.

### Overcoming tumour antigen escape

The development of novel CD3+ T-cell redirectors with the ability to target two tumour antigens is a potential strategy that can be used to overcome tumour antigen escape and restore anti-tumour immunity.^67^ In fact, the strategy of simultaneously targeting two tumour antigens using bispecific tandem CARs has already been demonstrated to mitigate tumour antigen escape.^68,69^ A single-nucleotide polymorphism (SNP) present in the V domain of CD33 is known to limit the anti-tumour activity of anti-CD33 antibodies due to antigen loss, but the development of a novel bispecific antibody that targets CD3 and the C2 domain of CD33 resulted in T-cell-mediated tumour lysis independent of the SNP genotype.^70^ These findings indicate a therapeutic benefit in designing CD3+ T-cell redirectors that target the C2 domain and not the V domain of the CD33 epitope for the treatment of AML.

### Improving tumour antigen specificity

Identifying antigenic targets that are specific to tumour cells and not expressed on healthy tissue is another challenge associated with developing CD3+ bispecific T-cell redirectors, especially for the treatment of solid malignancies. Even low-level antigen expression on normal cells can lead to detrimental on-target off-tumour toxicities in which the cytolytic activity of CD3+ T cells is directed towards healthy tissue. Preclinical studies suggest that tuning the affinity of CD3+ bispecific T-cell engagers towards target antigens is a rational approach to achieve an optimal balance of anti-tumour activity and safety.^71^ To reduce the occurrence of T-cell autoreactivity towards antigen-expressing vital organs, a multivalent avidity-based antigen-binding design strategy has been developed to generate an anti-CD3 × anti-HER2 bispecific antibody capable of selectively killing HER2-expressing tumour cells.^72^ Selective redirection of CD3+ T cells towards HER2+ cancer cells was achieved by engineering the anti-CD3 × anti-HER2 bispecific antibody with two low-affinity anti-HER2 Fab arms that engage HER2-overexpressing tumour cells with a high antigen density relative to healthy tissue. This seminal work provides proof of concept for redirecting the cytotoxic activity of T cells to tumour cells while sparing normal tissue and could be applicable to other solid tumour targets that are challenged by on-target off-tumour toxicity.

Another means to address the lack of tumour antigen specificity associated with CD3+ T-cell redirection involves the use of a conditionally active COBRA (**CO**nditional **B**ispecific **R**edirected **A**ctivation) T-cell engager platform. COBRAs are prodrugs that are selectively activated upon cleavage by matrix metalloproteases (MMPs) in the protease-rich TME.^73^ This selective activation enables them to remain as inactive prodrugs in normal tissue where the proteolytic activity is tightly regulated but become active T-cell engagers in the context of tumours, where proteases are ubiquitously expressed. Indeed, COBRA constructs with an MMP-9 cleavable linker targeting anti-CD3, anti-EGFR and anti-human serum albumin were shown to induce tumour regression in a xenograft mouse model of colorectal cancer.^73^ Additional protease-activated T-cell engagers have also been described. Tumour specificity was demonstrated using an anti-folate receptor 1 (FOLR1) T-cell bispecific antibody engineered with an anti-idiotypic anti-CD3 ‘mask’ joined to an anti-CD3 Fab region via a protease-cleavable linker. ‘Unmasking’ of the anti-CD3 targeting moiety by cleavage by active proteases in the TME led to selective killing of FOLR1-expressing tumour cells in vitro and in vivo, while sparing normal cells with low FOLR1 expression.^74^ Next-generation designs of masked antibodies that are capable of being selectively unmasked at tumour sites have been optimised. Incorporation of a heterodimeric coiled-coil domain into an antibody construct was shown to be an effective and generalisable approach for masking the complementary-determining regions (CDRs) of an antibody, with selective cleavage of coiled-coil peptides by tumour-associated proteases restoring antigen binding while overcoming on-target off-tumour toxicity.^75^ Moreover, preclinical proof-of-concept studies demonstrated that such probody therapeutics improved the safety profile of EGFR-targeting antibodies and broadened the therapeutic index.^76^ A first-in-human Phase 1/2 clinical trial evaluating the safety and tolerability of a PD-L1 probody therapeutic (CX-072) as monotherapy or in combination with ipilimumab or vemurafenib for the treatment of solid tumours or lymphomas (NCT03013491) is currently underway.

In addition to protease-activated T-cell engagers, a novel split T-cell engaging antibody format—referred to as a hemibody (e.g., an antigen-specific scFv fused to the VH or VL region of an anti-CD3 antibody)—has been described. Simultaneous engagement of two complementary hemibodies with their respective antigens on a single target cell activated the CD3-binding site and redirected CD3+ T cells for tumour cell lysis. Using this approach, hemibodies can selectively kill dual-antigen-positive tumour cells while sparing cells that express a single antigen, thereby overcoming the limitation of off-target toxicity commonly associated with CD3+ T-cell redirection.^77^

## Suboptimal potency

Improving the potency of CD3+ bispecific T-cell redirectors is an ongoing challenge. Both the size of the target antigen and the distance of the target epitope to the T-cell membrane have been identified as important factors contributing to the anti-tumour activity of CD3+ T-cell engagers.^78,79^ Interestingly, anti-CD3 × anti-chondroitin sulphate proteoglycan 4 (CSPG4) bispecific antibodies demonstrated greater potency, in terms of T-cell-mediated tumour lysis, when the size of the target antigen was smaller. Moreover, binding of the anti-CD3 × anti-CSPG4 bispecific antibody to the membrane-proximal domain D3 of CSPG4 rather than the distal domain elicited a more potent anti-tumour immune response by the redirected T cells.^78^ Additional factors, such as the copy number and valency of tumour antigens, have also been shown to affect the potency of bispecific T-cell redirectors.^79^ Collectively, these preclinical data suggest that awareness of target antigen size/copy number and modulation of binding domain positioning and valency should be considered in the design of more potent, best-in-class CD3+ T-cell redirectors.

## Concluding remarks and future directions

Antibody-based therapy—particularly CD3+ bispecific T-cell redirection—has revolutionised the field of cancer immunotherapy.^80^ From a preclinical aspect, anti-tumour activity in both haematological malignancies and solid cancers has been achieved with CD3+ T-cell engaging bispecific antibodies by addressing some of the major obstacles inherent to this therapeutic class, such as the expression of inhibitory immune checkpoint molecules, the presence of an immunosuppressive TME, suboptimal potency, escape of tumour antigens, cytokine storm and on-target off-tumour toxicity. Encouragingly, many of these novel CD3+ bispecific T-cell antibodies have progressed to clinical trials and next-generation designs of CD3+ bispecific T-cell redirectors are being characterised.

**Fig. 3:**
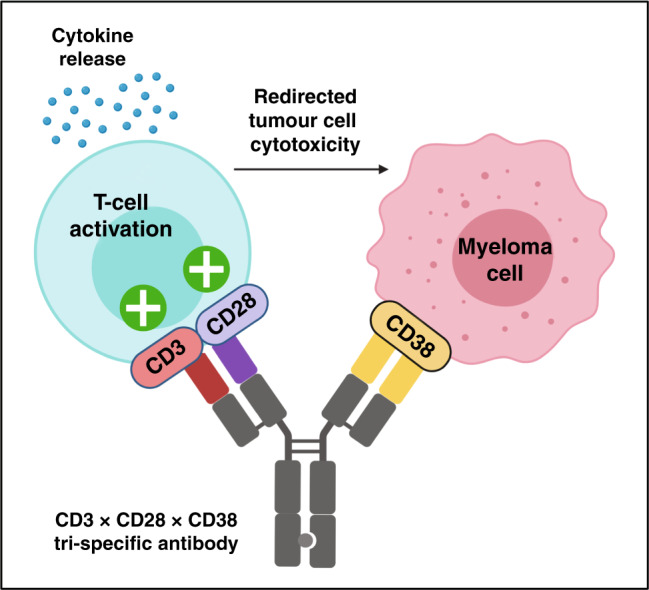
Next-generation anti-CD3 × anti-CD28 × anti-CD38 tri-specific T-cell-engaging antibody. The diagram depicts a tri-specific antibody targeting anti-CD3ε and anti-CD28 on T cells as well as anti-CD38 on multiple myeloma cells. Co-engagement of CD3, CD28 and CD38 results in efficient T-cell stimulation and activation, cytokine release, and redirection of T-cell-mediated cytotoxicity to myeloma cells.

Intriguingly, preclinical evidence has demonstrated that in vitro BiTE-mediated tumour cell cytotoxicity is enhanced by the expression of CD80/CD86 co-stimulatory molecules on tumour cells.^81,82^ The engagement of CD80/CD86-activating ligands on cancer cells with their cognate receptor (CD28) on naive T cells provides co-stimulation to support T-cell activity.^83^ These data are further corroborated by robust evidence suggesting that CAR-T cell potency is enhanced upon the incorporation of co-stimulatory domains (e.g., CD28, 4-1BB) into the CAR backbones.^84^ However, although co-stimulation via CD28 has provided benefit to BiTEs and CAR-T cells alike by enhancing anti-tumour activity without inducing toxicity, cases have arisen where too much co-stimulation has induced life-threatening immune-related adverse events such as cytokine storm, as observed using the anti-CD28 super-agonist TGN1412.^24^ As such, optimising co-stimulatory signalling is important for achieving anti-tumour activity while minimising toxicity. Accordingly, simultaneous multiple interaction T-cell-engaging (SMITE) bispecific antibodies, comprising a combination of two BiTE antibodies, have been developed.^85^ In fact, T-cell co-stimulation with an anti-CD28 × anti-ROR1 BiTE was shown to enhance the anti-tumour activity of a CD3 ×  ROR1 BiTE in K562 cells transduced with ROR1. In addition, co-activation of T cells with a CD28 × PD-L1 BiTE and a paired CD3 × CD19 BiTE was demonstrated to overcome BiTE resistance by reversing immune checkpoint inhibition in PD-L1-expressing cancer cells.^85^ Furthermore, a bispecific antibody retargeted CAR, comprising an extracellular folate receptor fused to intracellular TCR and CD28 costimulatory domains in tandem, has been developed.^86^ This work demonstrated proof of concept for combining bispecific antibodies with genetically engineered T cells, which led to simultaneous TCR activation/co-stimulation and induced a tumour-antigen-specific T-cell response. CD3+-redirecting tri-specific antibodies have also been designed to bridge T cells to tumour cells in the presence of co-stimulation. For example, the anti-CD3 × anti-CD28 × anti-CEA tri-specific antibody was shown to mediate tumour-specific cytolysis of CEA-expressing cancer cells in vitro.^87^ Along the same lines, an anti-CD3 × anti-CD28 × anti-CD38 tri-specific T-cell redirector antibody has been developed (Fig. 3), which, when administered in vivo, led to the proliferation of effector/memory T-cell populations and the suppression of tumour growth in CD38-expressing melanoma.^88^ Next-generation CD3+ T-cell engagers that exploit signalling through co-stimulatory molecules appear to represent promising antibody therapeutics for cancer immunotherapy.

A novel T-cell redirection strategy called STAb immunotherapy, which involves the endogenous **s**ecretion of **T** cell-redirecting bs**Ab**s (bispecific antibodies), has also emerged as a promising platform in oncology.^89^ In this approach, T cells are genetically engineered to secrete bispecific T-cell-redirecting antibodies intratumourally (known as ‘on-tumour’ STAbs) or at a distant site (‘off-tumour’ STAbs). This platform addresses some of the challenges associated with systemic antibody administration, such as inadequate tumour penetration, on-target off-tumour toxicity, and short serum half-life. Proof of concept for STAb cancer immunotherapy has been demonstrated in preclinical in vitro and in vivo studies.^90–92^

Overall, substantial progress has been made towards the development of CD3+ bispecific T-cell redirectors for cancer immunotherapy. A diverse panel of haematological and solid tumour targets have been characterised for CD3+ T-cell redirection, and the safety and efficacy of ~100 antibodies in this therapeutic class are currently under investigation in the clinic for the treatment of haematological cancers and solid tumours (Tables 2 and 3). Nonetheless, a number of challenges associated with CD3+ T-cell redirection exist that can limit anti-tumour efficacy. As many of these challenges are common to other cancer immunotherapy approaches such as CAR-T cell therapy,^93^ lessons learned from these studies can potentially be applied to CD3+ T-cell redirection. Future directions in the field of cancer immunotherapy are likely to involve identifying optimal combination therapies with CD3+ bispecific T-cell redirectors that enhance anti-tumour immunity while minimising toxicity.

**Table 2:** Clinical trials of CD3+ bispecific T-cell redirectors for haematological cancers.

Targets	Clinical trial Phase	Clinical trial identifier	Disease indication(s)	Organisation(s)	Antibody
CD3 × CD19	Launched	NCT01207388	B-cell precursor ALL	Amgen	Blinatumomab; Blincyto; AMG 103
CD3 × CD19	Phase 1	NCT03571828	B-cell NHL; DLBCL; MCL; FL	Amgen	AMG 562
CD3 × CD19	Phase 1	NCT04056975	B-cell lymphoma	Generon	A-319
CD3 × CD19	Phase 1	CTR20191955	B-cell lymphoma	Lvzhu Biopharmaceutical	K193
CD3 × CD20	Phase 1/2	NCT02651662 NCT02290951 NCT03888105	B-cell NHL; CLL	Regeneron	REGN1979
CD3 × CD20	Phase 1	NCT04082936	B-cell NHL; DLBCL; MCL; FL; MZL	IGM Biosciences	IGM-2323
CD3 × CD20	Phase 1	NCT02924402	B-cell NHL; CLL; small lymphocytic lymphoma	Xencor	Plamotamab; XmAb13676
CD3 × CD20	Phase 1/2	NCT02500407 NCT03671018 NCT03677141 NCT03677154	B-cell NHL; CLL; DLBCL	Genentech; Hoffmann-La Roche	Mosunetuzumab; BTCT4465A; RO7030816
CD3 × CD20	Phase 1/2	NCT03625037	DLBCL; MCL; FL	Genmab	GEN3013
CD3 × CD20	Phase 1	NCT03075696 NCT03533283 NCT04313608 NCT04246086 NCT04077723 NCT03467373	B-cell NHL; DLBCL; FL	Hoffmann-La Roche	Glofitamab; RO7082859
CD3 × CD20	Phase 1	NCT00244946 NCT00938626	B-cell NHL; multiple myeloma; plasma cell neoplasm	Barbara Ann Karmanos Cancer Institute	CD20Bi-ATC
CD3 × CD33	Phase 1	NCT03915379	Myelodysplastic syndrome; AML	Janssen	JNJ-67571244
CD3 × CD33	Phase 1	NCT02520427	Myelodysplastic syndrome; AML	Amgen	AMG 330
CD3 × CD33	Phase 1	NCT03224819	AML	Amgen	AMG 673
CD3 × CD33	Phase 1	NCT03516760	AML	Celgene	GEM333
CD3 × CD33	Phase 1	NCT04128423 NCT03144245 NCT03516591	Myelodysplastic syndrome; AML	Amphivena Therapeutics	AMV564
CD3 × CD38	Phase 1/2	NCT03309111	Multiple myeloma	Glenmark Pharmaceuticals	GBR 1342
CD3 × CD38	Phase 1	NCT03445663	Multiple myeloma	Amgen	AMG 424
CD3 × CD123	Phase 1	NCT03647800	Myelodysplastic syndrome; AML	Aptevo Therapeutics	APVO436
CD3 × CD123	Phase 1/2	NCT04158739 NCT02152956 NCT03739606	AML; ALL; other haematologic malignancies	MacroGenics	Flotetuzumab; MGD006
CD3 × CD123	Phase 1	NCT02730312	B-cell ALL; AML; CLL; blastic plasmacytoid dendritic cell neoplasm	Xencor	Vibecotamab; XmAb14045
CD3 × CD123	Phase 1/2	NCT03594955	B-cell ALL; AML; myelodysplastic syndrome	Sanofi	SAR440234
CD3 × CD123	Phase 1	NCT02715011	AML	Janssen	JNJ-63709178
CD3 × BCMA	Phase 1	NCT02514239 NCT03836053	Multiple myeloma	Boehringer Ingelheim; Amgen	BI 836909; AMG 420
CD3 × BCMA	Phase 1	NCT03287908	Multiple myeloma	Amgen	AMG 701
CD3 × BCMA	Phase 1	NCT03933735	Multiple myeloma	AbbVie; TeneoBio	TNB-383B
CD3 × BCMA	Phase 1	NCT03269136	Multiple myeloma	Pfizer	PF-06863135
CD3 × BCMA	Phase 1	NCT03145181 NCT04108195	Multiple myeloma	Janssen	JNJ-64007957
CD3 × BCMA	Phase 1	NCT03486067	Multiple myeloma	Celgene	CC-93269
CD3 × BCMA	Phase 1/2	NCT03761108	Multiple myeloma	Regeneron; Sanofi	REGN5458
CD3 × BCMA	Phase 1	NCT04083534	Multiple myeloma	Regeneron; Sanofi	REGN5459
CD3 × BCMA × ALB	Phase 1/2	NCT04184050	Multiple myeloma	Harpoon Therapeutics; AbbVie	HPN217
CD3 × CLEC12A	Phase 1	NCT03038230	AML	Merus	Tepoditamab; MCLA-117
CD3 × FcRH5	Phase 1	NCT03275103	Multiple myeloma	Genentech	RO7187797; BFCR4350A
CD3 × FLT3	Phase 1	NCT03541369	AML	Amgen	AMG 427
CD3 × GPRC5D	Phase 1	NCT03399799 NCT04108195	Multiple myeloma	Janssen	JNJ-64407564

*BCMA* B-cell maturation antigen, *ALB* albumin, *CLEC12A* C-type lectin domain family 12 member A, *FcRH5* Fc receptor-homology 5, *FLT3* Fms-like tyrosine kinase 3, *GPRC5D* G protein-coupled receptor class C group 5 member D, *AML* acute lymphoblastic leukaemia, *NHL* non-Hodgkin lymphoma, *DLBCL* diffuse large B-cell lymphoma, *MCL* mantle cell lymphoma, *FL* follicular lymphoma, *CLL* chronic lymphocytic leukaemia, *MZL* marginal zone lymphoma, *AML* acute myeloid leukaemia.

**Table 3:** Clinical trials of CD3+ bispecific T-cell redirectors for solid cancers.

Targets	Clinical trial Phase	Clinical trial identifier	Disease indication(s)	Organisation(s)	Antibody
CD3 × B7-H3	Phase 1	NCT03406949	Advanced solid tumours	MacroGenics	Orlotamab; MGD009
CD3 × CEACAM	Phase 1	NCT02291614 NCT01284231 NCT02760199	Gastrointestinal adenocarcinoma	Amgen; MedImmune	AMG 211; MEDI-565
CD3 × CEACAM	Phase 1/2	NCT03866239 NCT03337698 NCT02650713 NCT02324257	Colorectal cancer; NSCLC	Hoffmann-La Roche	Cibisatamab; RO6958688
CD3 × CLDN18.2	Phase 1	NCT04260191	Gastric and gastro-oesophageal junction adenocarcinoma	Amgen	AMG 910
CD3 × DLL3	Phase 1	NCT03319940	SCLC	Amgen	AMG 757
CD3 × EGFRvIII	Phase 1	NCT03296696	Glioblastoma	Amgen	AMG 596
CD3 × EGFR	Phase 1/2	NCT01420874 NCT02620865 NCT03269526 NCT03344250	Pancreatic cancer; colorectal cancer; glioblastoma	Barbara Ann Karmanos Cancer Institute; University of Virginia	EGFR2Bi-ATC; EGFR-BATs
CD3 × EpCAM	Phase 1	CTR20181212	Malignant ascites	Wuhan YZY Biopharma	M701
CD3 × EpCAM	Withdrawn from market	Withdrawn from market	Malignant ascites	Trion Pharma	Catumaxomab; Removab
CD3 × GD2	Phase 1/2	NCT02173093	Neuroblastoma	University of Virginia	GD2Bi-aATC
CD3 × gpA33	Phase 1/2	NCT03531632 NCT02248805	Colorectal cancer	MacroGenics	MGD007
CD3 × gp100	Phase 1/2	NCT03070392 NCT02535078 NCT02570308 NCT01211262 NCT01209676	Uveal melanoma; malignant melanoma	Immunocore; University of Pennsylvania	IMCgp100
CD3 × GPC3	Phase 1	NCT02748837	Solid tumours	Chugai Pharmaceutical	ERY974
CD3 × GUCY2c	Phase 1	NCT04171141	Gastrointestinal tumours; colorectal cancer; oesophageal adenocarcinomas	Pfizer	PF-07062119
CD3 × HER2	Phase 1/2	NCT03406858 NCT03661424 NCT03272334 NCT00027807	Prostate cancer; breast cancer; leptomeningeal metastases	Barbara Ann Karmanos Cancer Institute; University of Virginia	HER2Bi-ATC; HER2-BATs
CD3 × HER2	Phase 1	NCT03448042	Solid tumours	Genentech	BTRC4017A
CD3 × HER2	Phase 1/2	NCT03983395	Breast cancer	Glenmark Pharmaceuticals	GBR 1302; ISB 1302
CD3 × HER2	Phase 1	CTR20171194	Advanced solid tumours	Wuhan YZY Biopharma	M802
CD3 × MAGE-A4	Phase 1/2	NCT03973333	Advanced solid tumours	Immunocore Ltd	IMC-C103C
CD3 × MSLN × ALB	Phase 1/2	NCT03872206	Ovarian cancer; fallopian tube cancer; peritoneal cancer; pancreatic cancer; mesothelioma	Harpoon Therapeutics	HPN536
CD3 × MUC1	Phase 2	NCT03540199 NCT03524261 NCT03501056 NCT03554395 NCT03509298 NCT03524274 NCT03484962 NCT03146637	Kidney cancer; breast cancer; lung cancer; gastric cancer; pancreatic cancer; colorectal cancer; liver cancer	Fuda Cancer Hospital, Guangzhou; Benhealth Biopharmaceutical	Activated CIK and CD3-MUC1
CD3 × MUC16	Phase 1/2	NCT03564340	Ovarian cancer; fallopian tube cancer; primary peritoneal cancer	Regeneron; Sanofi	REGN4018
CD3 × MUC17	Phase 1	NCT04117958	Gastric and gastro-oesophageal junction cancer	Amgen	AMG 199
CD3 × P-cadherin	Phase 1	NCT02659631	Triple-negative breast cancer; colorectal cancer; NSCLC	Pfizer	PF-06671008
CD3 × PRAME	Phase 1/2	NCT04262466	Advanced solid tumours	Immunocore Ltd	IMC-F106C
CD3 × PSCA	Phase 1	NCT03927573	Pancreatic cancer; breast cancer; urogenital cancer; NSCLC	Celgene	GEM3PSCA
CD3 × PSMA	Phase 1	NCT03926013	Prostate cancer; renal cell carcinoma	Janssen	JNJ-63898081
CD3 × PSMA	Phase 1	NCT03792841	Prostate cancer	Amgen	AMG 160
CD3 × PSMA	Phase 1	NCT01723475	Prostate cancer	Amgen; Bayer	Pasotuxizumab; AMG 212
CD3 × PSMA	Phase 1	NCT02262910	Prostate cancer	Aptevo Therapeutics	ES414
CD3 × PSMA × ALB	Phase 1	NCT03577028	Prostate cancer	Harpoon Therapeutics	HPN424
CD3 × SSTR2	Phase 1	NCT03411915	Neuroendocrine tumours; gastrointestinal stromal tumours	Xencor	Tidutamab; XmAb18087
CD3 × STEAP1	Phase 1	NCT04221542	Prostate cancer	Amgen	AMG 509
CD3 × 5T4	Phase 1/2	NCT04424641	Solid tumours	Genmab; AbbVie	GEN1044

*CEACAM* carcinoembryonic antigen-related adhesion molecule, *CLDN18.2* claudin-18 isoform 2, *DLL3* delta-like canonical notch ligand 3, *EGFRvIII* epidermal growth factor receptor vIII, *EGFR* epidermal growth factor receptor, *EpCAM* epithelial cell adhesion molecule, *gpA33* glycoprotein A33, *gp100* glycoprotein 100, *GUCY2c* guanylate cyclase 2c, *HER2* human epidermal growth factor receptor 2, *MAGE-A4* melanoma-associated antigen A4, *MSLN* mesothelin, *ALB* albumin, *MUC1* mucin 1, *MUC16* mucin 16, *MUC17* mucin 17, *PRAME* preferentially expressed antigen in melanoma, *PSCA* prostate stem cell antigen, *PSMA* prostate-specific membrane antigen, *SSTR2* somatostatin receptor type 2, *STEAP1* six transmembrane epithelial antigen of the prostate 1, *NSCLC* non-small cell lung cancer, *SCLC* small-cell lung cancer.

## Data Availability

Not applicable.
